# In silico design and in vitro development of a highly accurate test to detect Brucella species

**DOI:** 10.3906/sag-1807-20

**Published:** 2019-02-11

**Authors:** Hosein SAFARI, Setareh SHAHNAZARI, Maysam MARD-SOLTANI, Somayeh TAVAKOLI, Bahman KHALESI

**Affiliations:** 1 Nano-Biotechnology Research Center, Baqiyatallah University of Medical Sciences, Tehran Iran; 2 Department of Biology, Payame Noor University, Tehran Iran; 3 Department of Clinical Biochemistry, Faculty of Medical Sciences, Dezful University of Medical Sciences, Dezful Iran; 4 Department of Virology, Pasteur Institute of Iran, Tehran Iran; 5 Department of Research and Production of Poultry Viral Vaccine, Razi Vaccine and Serum Research institute,Agricultural Research Education and Extension Organization (AREEO), Karaj Iran

**Keywords:** Brucellosis, diagnosis, Omp2, primer design, multiple sequence alignment, PCR

## Abstract

**Background/aim:**

Conventional methods of detecting Brucella spp. suffer from technical and biological complications. Besides, newly characterized species of the genus Brucella could be neglected by previously designed polymerase chain reaction (PCR) tests. Therefore, a more accurate PCR-based test seems to be imminently needed.

**Materials and methods:**

Blood samples were collected from 39 patients diagnosed with brucellosis and 25 healthy controls. Multiple sequence alignments (MSA) were performed on 500 Omp2-related protein and gene sequences. Thereafter, specific primers were designed and synthesized for the regions with highest conservancy. The collected samples were assessed by PCR test. To overcome the cross-reactivity issue, PCR thermal program was optimized regarding annealing time and temperature.

**Results:**

The MSA results indicated that the N terminus region of the Omp2 protein (DNA 5’ end) is associated with highest conservancy. Primers with highest specificity were designed and synthesized. A two-step PCR reaction was successfully designed and optimized. The desirable bands were observed in clinical samples with high accuracy.

**Conclusion:**

It should be pointed out that using a precisely designed primer pair would bring about early infection detection, more success to detect all natural variants and higher cost-to-efficacy ratio in comparison to other detection methods.

## 1. Introduction

Brucellosis is a highly contagious zoonosis infection caused by *Brucella* spp. which are gram-negative, aerobic, nonsporulating, and coccobacillus bacteria. This infection (also known as Malta fever) is common in human and some animal species (1). More than 300 million cattle infections (out of the 1.4 billion cattle population worldwide), as well as more than 500,000 annual new human infections, are estimated to be the global disease burden for brucellosis (2). David Bruce was the first to identify the relationship between these bacteria and the resulting disease in 1887. During 1905, Themistocles Zammit discovered that consumption and/or close contact to unsterilized milk and meat of contaminated animals is the source of this infection. The members of the genus *Brucella* are facultative intracytoplasmic parasites traditionally consisting of 6 recognized species. *Brucella melitensis*, *Brucella abortus*, *Brucella suis*, *Brucella canis*, *Brucella ovis*, and *Brucella neotomae* are respectively responsible for pathogenesis in goats and sheep, cows, pigs, dogs, sheep, and pack rat midden (*Neotoma cinerea*). However, *B. inopinata*, *B. pinnipedialis*, *B. ceti*, and *B. microti* are newly characterized species of the genus *Brucella* isolated from human, aquatic mammals, and common vole, respectively (3). The failure of attempts to eradicate this disease and the absence of any approved human vaccines underscores the significance of this infection as an ongoing clinical issue.

Diagnostic approaches for identification of brucellosis include microbiological, serological, and molecular approaches. Analyzing blood, bone marrow, cerebrospinal fluid, and synovial fluid (during fevers) samples along with the screening of culture isolates are the common ways of *Brucella* detection. Unfortunately, sample culture and screening is not achievable in chronic infections. World Health Organization (WHO) has described 5 standard serologic experiments to identify the *Brucella* infection. These experiments include: Rose Bengal, Sero-agglutination or Wright, 2 Mercaptoethanol–Wright, Complement Fixation, and Antiglobulin or Coombs–Wright which are all dependent on different levels of human immune responses against the pathogen. Complexity of pathogenicity and chronic states along with *B. abortus* and *B. canis* infection reduces the blood culture-based identification chances to 30%–50%. Long life cycles (more than 30 days) of *Brucella* spp. make golden tests inappropriate (4). Aforementioned serological tests with 65%–95% diagnostic accuracy not only are inappropriate in endemic regions with high frequency of antibody existence but also have cross-reactivity with other bacteria (5). Different aspects of *Brucella* spp. genomes have already been studied. Availability of DNA, RNA, and ribosomal RNA (rRNA) of *Brucella* spp. in human blood makes it amenable for molecular detection via PCR-based methods. The DNA sequences of 43 kDa membrane protein of *B. aburtus* (6) and 31 kDa BCSP31 of *B. aburtus* and *B. melitensis* (7) were the first sequences employed for *Brucella* spp. detection using PCR-based molecular techniques. Various ribosomal DNA (rDNA) regions such as 16S gene or fragments between 16S and 23S genes were also analyzed with different PCR techniques (8–10). 

Although molecular methods of *Brucella* spp. detection have some advantages over conventional microbiological and serological methods, low sensitivity could be the only cause of their poor detection ability. This could be the consequence of natural polymorphisms or newly characterized species whose sequences were not taken into account in PCR test design. Bioinformatics approaches are now widely used in various fields of biology (11–18). Using bioinformatics tools could be a compelling approach to circumvent the sensitivity issues of the molecular methods of *Brucella* spp. detection. In the present study, we exploited bioinformatics tools to analyze the sequences of Omp2 gene from *Brucella* spp. to identify the regions with highest conservancy and design-specific primers with highest convergence for *Brucella *Omp2 gene sequences. To evaluate the applicability of the designed PCR test, infected samples were collected from Hamedan and Bahar cities and used for possible improved molecular detection.

## 2. Materials and methods

### 2.1. Patients and specimens 

Samples were collected from hospitalized patients with brucellosis diagnosed by antibody raises (1/320-1/1280) from Hamedan and Bahar cities. Overall, 29 blood samples were obtained by venipuncture and used for further studies. Top Bioscience genomic extraction kit (Iran) was employed to extract DNA contents. Briefly, 200 µL of blood sample and 20 µL proteinase K were gently mixed and centrifuged for 5 min at 400 *g*. The pellets were resuspended in 200 µL of PBS. 200 µL of lysis buffer was added into the solution and incubated at 20° C for 30 min. The solution was then treated by 200 µL of ice-cold pure ethanol by gentle pipetting. The solution was later centrifuged (MERCK, Germany) at 6000 rpm for 1 min in DNA extraction kit columns (Top BioScience, Iran). Washing and elusion buffers were added subsequently after each centrifuge and microtube replacement. DNA concentration in TAE buffer (BIOIDEA, Iran) was determined with NanoDrop spectrophotometer (2000c, Thermo Fisher Scientific, USA).

### 2.2. Conservancy analyses and Brucella-specific primer design

The sequence of Omp2 gene and protein from *Brucella abortus* was obtained from NCBI gene bank and protein database at https://www.ncbi.nlm.nih.gov. The Omp2 DNA and protein sequences were used to perform nucleotide and protein basic local alignment search tool (BLAST) searches at https://blast.ncbi.nlm.nih.gov/Blast.cgi. The BLAST searches were limited to the *Brucella* spp. sequences. The number of target sequences to be returned was set to 500 sequences. The sequences of Omp2 from different *Brucella* species were aligned with MEGA software (version 7) for both DNA and protein sequences. The conserved regions of the Omp2 gene and protein were recognized using alignment results. The BioEdit software was employed to draw entropy plots of Omp2 DNA and protein alignment results. Primers were designed by Primer3 Online program (http://primer3.sourceforge.net). Thermodynamic characteristics of the primers, such as stability, dimer, or loop formation, were analyzed with Allele ID software (version 6) and Gene Runner software (version 3.05). Approved primer pairs were used to perform primer BLAST search at https://www.ncbi.nlm.nih.gov/tools/primer-blast/. 

### 2.3. Preparation of internal control (IC)

A previously described set of primer pair was selected to be employed as the IC gene. The primer is specific to the chromosome X of Drosophila melanogaster (19). The resulting PCR product was cloned into a common TA vector. The Thermo Fisher Scientific TA cloning kit was employed to perform the cloning. All procedures were carried out according to the instructions of the manufacturer. The final plasmid was transformed into chemically competent *E. coli* bacteria. DNA sequencing was finally used to confirm the procedure and the accuracy of the cloned sequences. Standard plasmid extraction kit (GeneAll Biotechnology, Korea) was employed to obtain the cloned TA vectors according to the manufacturer’s instructions. Nanodrop spectrophotometer (2000c, Thermo Fisher Scientific, USA) was employed to calculate the concentration of the extracted plasmids. 

### 2.4. PCR test setup

PCR tests were designed to set the best conditions of reactions for IC, reference strain of *Brucella abortus* (purchased from the reference laboratory of Iran and the Pasteur Institute of Iran) and coreactions of IC and reference sequence. Different optimization procedures were performed to improve PCR conditions regarding the temperatures and durations of each PCR step. PCR products were analyzed by agarose gel electrophoresis (Invitrogen, USA) along with 100 bp DNA Marker (Cinagen, Iran) in TBE (Bio-Idea, Iran) buffered electrophoresis tank (Biorad, USA). Results were visualized in a gel documentation device (UVSAVE HD2, UVITEC, ENGLAND) after staining with GelRed (BIOTUM, USA).

## 3. Results

### 3.1. Sequence analyses

**Figure 1:**
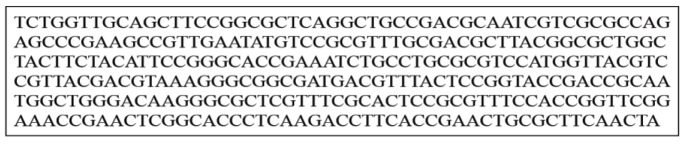
The sequence of the conserved region of the Omp2 gene.

**Figure 2:**
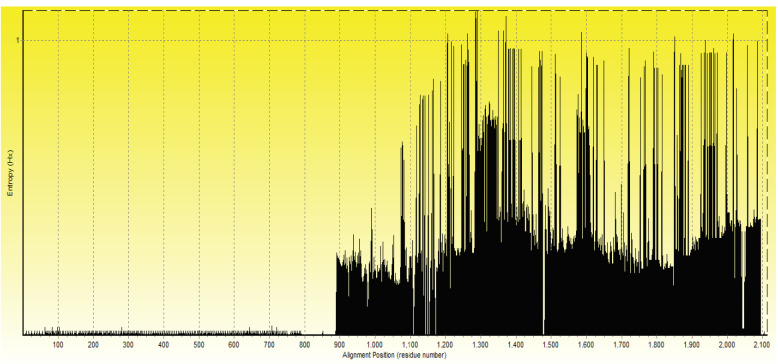
The entropy plot of Omp2 gene sequences derived from multiple sequence alignment (MSA) results. The first 1100 positions are the sequences with lowest diversity. This region includes the 300 base pair (bp) of the 5’ end of Omp2 gene. The existing gaps in the MSA results extended the length of the plot.

The reference sequence of *Brucella abortus* Omp2 gene was obtained under the NCBI ID of NC_006932.1. The Omp2 gene is a 1098 nucleotide gene which is translated into a 362 amino acids protein. The BLAST search results lead to identification of 500 sequences with high sequence similarity to Omp2 gene and protein. The BLAST search results included sequences from all 10 *Brucella* spp.. The alignment results for different Omp2 genes and proteins showed that there is a highly conserved region at the N terminus region of the Omp2 protein, while the rest of the sequence is more variable. This region includes 100 amino acids of the protein N terminus. These observations were confirmed with the results of the Omp2 gene MSA. It has been shown that initial 300 bp form the 5’ end of Omp2 gene (Figure 1) is the best fit for the primer design analyses. Moreover, our entropy plot calculations have confirmed the results of protein and DNA MSAs (Figure 2). 

### 3.2. Primer design

The analyses for primer design require 2 pairs of primers for IC and Omp2 genes. The primer sequences for the genes are listed in Table 1. The primer BLAST analyses we performed demonstrated no cross-reactions between Omp2 and other *Brucella* genes as well as human; and their specificity was confirmed for Omp2a and 2b.

**Table 1:** Primer pairs for internal control (IC) and Brucella polymerase chain reaction (PCR) process along with their product sizes.

Name of primer	Sequence of primer	Product size
Brucella froward	CGAAGCCGTTGAATATGTCC	233 bp
Brucella reverse	GTTCGGTGAAGGTCTTGAGG	
IC froward	AGCATTCAAATCCTTCATACTG	684 bp
IC reverse	ATGTTGGTGTAATCTGACTCG	

### 3.3. Gene cloning and PCR reaction setup

The sequence of IC was successfully inserted into TA vector. The sequencing results confirmed the accuracy of the cloning process. The concentrations of extracted DNA was 100 and 40 ng/µL for IC containing TA vector and genomic DNA extracted from clinical samples and reference strain of *Brucella*. The accuracy of PCR amplification was confirmed for *Brucella* using standard bacteria. The volumes of each PCR ingredient is listed for single and coreaction PCR conditions in Table 2. The initial PCR program for single and coreaction PCRs was as follow:

**Table 2:** The volumes of samples employed for PCR are listed for single and coreactions.

Component	Volume for IC	Volume for Brucella	Volume for coreactions	Conc.
Taq 2X master mix	25 µL	25 µL	25 µL	1X
Primer F	1 µL	1 µL	1 µL	200 nM
Primer R	1 µL	1 µL	1 µL	200nM
Distilled water	21 µL	18 µL	16 µL	----
IC DNA	2 µL	-	2 µL	200 ng
Brucella DNA	-	5 µL	5 µL	200 ng
Total Volume	50 µL	50 µL	50 µL	----

{[Initial denaturation: 95 °C for 5 min][35 cycles of (denaturation: 95 °C for 20 s, annealing: 60 °C for 30 s, extension: 72 °C for 30 s)][Final extension: 72 °C for 7 min]}

This reaction condition could optimally amplify the single PCRs for the IC and reference *Brucella *genome. However, in the case of samples from patients, extra settings were needed. Although the specific band was observed (233 bp) at annealing temperature of 60 °C, unspecific bands were also traced in the vicinity of coreactions for clinical samples. Increasing number of reaction cycles into 40 and Touchdown PCR with 200 and 140 nM concentrations of *Brucella* primers could not eliminate nonspecific bands. Finally, two-step PCR was used based on following PCR protocol and thermal program:

{[Initial denaturation: 95 °C for 5 min] [10 cycles of (denaturation: 95 °C for 45 s, annealing: 57 °C for 30 s, extension: 72 °C for 10 s)] [Fin.Ext:72 °C for 2 min]}c{25 cycles of (denaturation: 95 °C for 25 s, annealing: 60 °C for 30 s, extension: 72 °C for 10 s)] [Final extension: 72°C for 2 min]}

**Figure 3:**
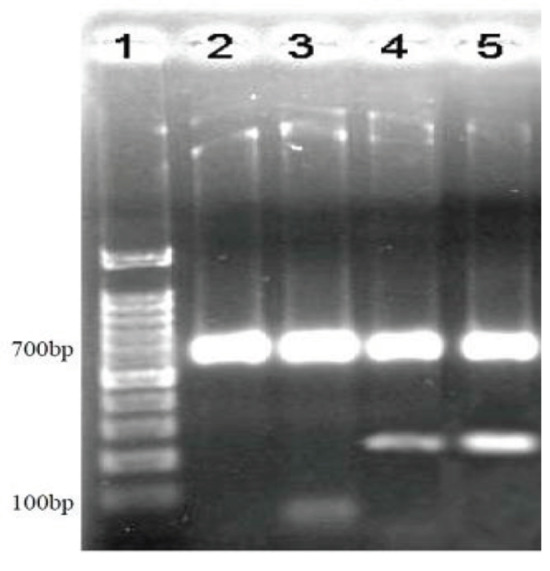
Coreaction of Brucella and internal control (IC) primers. Lane 1 is 100 bp DNA ladder, lanes 2 and 3 are PCR products of two positive clinical samples before optimization, lanes 4 and 5 are PCR products of the same samples after optimization. The 233 bp band appeared after optimization of coreaction PCR conditions.

The two-step program was successful at amplifying both IC and *Brucella* Omp2 DNA bands (Figure 3). 

### 3.4. PCR for clinical samples

Optimized PCR reaction was done on 39 samples from patients with brucellosis and 25 healthy people. Intriguingly, the PCR reactions correctly developed a 233 bp DNA band for all samples from patients, while the healthy samples only had the 684 bp band for IC. This means that the PCR reaction is able to detect the *Brucella* spp. with high accuracy.

## 4. Discussion

*Brucella* spp. are currently detected by microbiological methods of bacterial culture from various biological samples, serological methods of detecting anti-*Brucella* antibodies, and molecular methods of detecting *Brucella* DNA. Microbiological methods are based on cultures from blood, tissue, and body fluids which suffer from low sensitivity (15%–70%), long incubation period, and a risk of laboratory-acquired infections (20). On the other hand, serological methods could fail in early disease detection due to their low sensitivity, possible serological cross-reactions, and inability to differentiate between the antibodies from an ongoing infection and persisting antibodies after therapy (21). However, PCR-based methods of *Brucella* spp. detection provide a sensitive and specific way for fast and accurate identification of this pathogen (7). In this regard, we have developed a PCR based method to detect *Brucella* species. Fekete et al. were the first to introduce a PCR-based test for detection of brucellosis (22). Their PCR reaction was based on the amplification a 635 bp fragment (outer membrane protein gene) from *B. abortus* strain 19. To the best of our knowledge, more than 200 studies have been conducted employing various PCR based methods to develop a useful tool for rapid brucellosis diagnosis. Various PCR-based methods including standard PCR, real-time PCR, multiplex PCR, nested and seminested PCR, and other PCR-based assays have already been exploited for detection of *Brucella* spp. (23). The attained progresses in PCR techniques provide a suitable means for specific identification of the infectious agents. However, there is no standard setting for proper molecular screening of *Brucella* infection. It should be noted that blood samples for PCR detection are vulnerable because of possible polymerase inhibitors like hemoglobin and human DNA of white blood cells (WBC) and the reaction conditions should be finely tuned for accurate detection. Although several factors have been reported to affect the PCR results for detection of* Brucella* spp., the efficiency of standard PCR tests mainly depend on specificity of the designed primers. 

The Omp2 gene is one of the highly preferred targets for PCR-based detection of* Brucella* species. Omp proteins are bacterial membrane porins which control osmotic pressure of bacterium. Fiche et al. (1994) discovered this gene and its polymorphisms for the first time (24). This locus contains two similar genes, Omp2a and Omp2b, which are separated with a 1900 bp fragment. *B. aburtus* has a 108 bp deletion in bioware 1 and 2, while *B. ovis* possesses two copies of Omp2a. *Brucella* isolates from aquatic mammalians lack Omp2b but has two copies of Omp2a (25). However, polymorphisms are often located at 3’ end of genes which makes 5’ end suitable for targeting. In 1995, Leal et al. targeted Omp2 gene with specific primers and it was proved to be more accurate than serologic tests for blood and milk samples of goats (26). This gene is reported to have a head-to-head duplication of open reading frame with 85% homology (27). Therefore, targeting this gene for PCR-based detection could result in two times the normal amount of DNA amplification. This property could be attained designing specific primers for conserved regions of these genes. The Omp2 locus of *Brucella* species and strains was reported to exhibit the highest degree of polymorphism (28). Baddour et al. reported that, among the three pairs of the evaluated primers, the primer pair for amplification of Omp2 gene is associated with the low sensitivity for detection of *Brucella* spp. from human blood samples (29). We believe that low sensitivity of Omp2-based detection of *Brucella* spp. could be rooted in the high polymorphism rate of this genomic location. However, this issue could be circumvented via designing specific primers for the regions of Omp locus with highest conservancy and highest coverage for sequences from all *Brucella* species. Such a primer pair could amplify the sequences with polymorphism and the newly characterized sequences. Performing a multiple sequence alignment for a large number of Omp sequences (including sequences with different polymorphisms and newly introduced sequences) is a prerequisite for such primer design. In this regard, based on multiple sequence alignment of 500 different Omp genes from *Brucella* spp., an important region of Omp2 gene (with highly conserved regions) was identified within the gene sequence. Our results for Omp2 gene proved equal or better accuracy compared to other molecular techniques for rapid detection of suspicious samples. Two-step PCR was utilized for accurate identification of mentioned specific sites. Some other studies are available with high accuracy and lower specificity based on rDNA of *Brucella* (7,30). Moreover, other PCR techniques such as nested PCR of fragments between 16S and 23S can improve the obtained results (31). 

In conclusion, it should be noted that PCR-based techniques could provide the healthcare professionals with more accurate information about the *Brucella* infection. Our setting could circumvent the drawbacks associated with conventional serum-based *Brucella* detection methods. Moreover, due to inclusion of various Omp2 sequences, the sensitivity of the designed PCR test would be higher than the previously described tests. Although, this gene could be analyzed with more accurate PCR hybrid techniques such as Loop-mediated Isothermal Amplification (LAMP), the higher cost of these methods makes our PCR-based approach more reasonable.
